# Supplementary material for: Epidemiological clustered characteristics of coronavirus disease 2019 (COVID-19) in three phases of transmission in Jilin Province, China
Source: PLoS One. 2023 Jan 19;18(1):e0279879. doi: 10.1371/journal.pone.0279879 (PMC9851530; doi:10.1371/journal.pone.0279879)
Supplement: S1 Table — (PDF) [file pone.0279879.s001.pdf]

The first epidemic phase

Case Number	The onset date	Date of diagnosis
1	2020/1/12	2020/1/23
2	2020/1/17	2020/1/25
3	2020/1/22	2020/1/24
4	2020/1/15	2020/1/24
5	2020/1/22	2020/1/27
6	2020/1/25	2020/1/27
7	2020/1/17	2020/1/28
8	2020/1/26	2020/1/28
9	2020/1/27	2020/1/29
10	2020/1/24	2020/1/30
11	2020/1/19	2020/1/30
12	2020/1/23	2020/1/30
13	2020/1/22	2020/1/30
14	2020/1/23	2020/1/30
15	2020/1/25	2020/2/1
16	2020/1/22	2020/2/1
17	2020/1/27	2020/2/1
18	2020/1/27	2020/2/2
19	2020/1/28	2020/2/2
20	2020/1/29	2020/2/2
21	2020/1/22	2020/2/2
22	2020/1/22	2020/2/2
23	2020/1/22	2020/2/2
24	2020/1/31	2020/2/3
25	2020/1/24	2020/2/3
26	2020/1/24	2020/2/3
27	2020/1/29	2020/2/3
28	2020/1/28	2020/2/3
29	2020/1/27	2020/2/3
30	2020/1/23	2020/2/3
31	2020/1/26	2020/2/4
32	2020/2/1	2020/2/4
33	2020/1/27	2020/2/4
34	2020/1/23	2020/2/4
35	2020/1/28	2020/2/4
36	2020/1/30	2020/2/4
37	2020/1/30	2020/2/4
38	2020/1/30	2020/2/4
39	2020/1/30	2020/2/4
40	2020/1/23	2020/2/4
41	2020/1/20	2020/2/4
42	2020/1/28	2020/2/5
43	2020/1/26	2020/2/5
44	2020/1/30	2020/2/5
45	2020/1/28	2020/2/5
46	2020/2/3	2020/2/5
47	2020/1/28	2020/2/5
48	2020/2/1	2020/2/5
49	2020/1/30	2020/2/5
50	2020/1/30	2020/2/5
51	2020/1/30	2020/2/5
52	2020/2/1	2020/2/5

53	2020/1/22	2020/2/5
54	2020/2/2	2020/2/6
55	2020/1/31	2020/2/6
56	2020/1/29	2020/2/6
57	2020/2/4	2020/2/6
58	2020/1/29	2020/2/6
59	2020/2/4	2020/2/7
60	2020/2/3	2020/2/7
61	2020/2/3	2020/2/7
62	2020/1/31	2020/2/7
63	2020/2/3	2020/2/7
64	2020/2/6	2020/2/7
65	2020/2/5	2020/2/8
66	2020/2/1	2020/2/8
67	2020/2/4	2020/2/8
68	2020/1/29	2020/2/8
69	2020/2/6	2020/2/9
70	2020/2/7	2020/2/9
71	2020/1/31	2020/2/9
72	2020/2/1	2020/2/9
73	2020/2/1	2020/2/9
74	2020/2/4	2020/2/9
75	2020/1/28	2020/2/9
76	2020/2/7	2020/2/9
77	2020/1/28	2020/2/9
78	2020/2/9	2020/2/10
79	2020/2/2	2020/2/10
80	2020/2/4	2020/2/11
81	2020/2/4	2020/2/12
82	2020/2/2	2020/2/12
83	2020/2/1	2020/2/13
84	2020/2/11	2020/2/14
85	2020/2/1	2020/2/14
86	2020/2/7	2020/2/15
87	2020/2/14	2020/2/15
88	2020/2/12	2020/2/16
89	2020/2/12	2020/2/19
90	2020/2/18	2020/2/20
91	2020/2/19	2020/2/24
92	2020/2/14	2020/2/24
93	2020/1/26	2020/2/3
94	2020/2/5	2020/2/5
95	2020/2/7	2020/2/7
96	2020/2/14	2020/2/14
97	2020/2/14	2020/2/14

	The second epidemic phase	
Case Number	the onset date	Date of diagnosis
1	2020/5/5	2020/5/10
2	2020/5/11	2020/5/14
3	2020/5/16	2020/5/16
4	2020/5/3	2020/5/9
5	2020/5/5	2020/5/9
6	2020/5/3	2020/5/23
7	2020/5/6	2020/5/9
8	2020/5/7	2020/5/9
9	2020/5/6	2020/5/9
10	2020/5/1	2020/5/9
11	2020/4/30	2020/5/12
12	2020/5/7	2020/5/9
13	2020/5/1	2020/5/9
14	2020/5/3	2020/5/10
15	2020/5/8	2020/5/10
16	2020/5/10	2020/5/12
17	2020/5/10	2020/5/12
18	2020/5/12	2020/5/12
19	2020/5/11	2020/5/12
20	2020/5/11	2020/5/12
21	2020/5/10	2020/5/12
22	2020/5/11	2020/5/13
23	2020/5/15	2020/5/16
24	2020/5/12	2020/5/17
25	2020/5/14	2020/5/17
26	2020/5/17	2020/5/18
27	2020/5/10	2020/5/10
28	2020/5/12	2020/5/14
29	2020/5/14	2020/5/15
30	2020/5/14	2020/5/16
31	2020/5/14	2020/5/17
32	2020/5/13	2020/5/14
33	2020/5/15	2020/5/18
34	2020/5/15	2020/5/18
35	2020/5/14	2020/5/18
36	2020/5/18	2020/5/19
37	2020/5/21	2020/5/21
38	2020/5/21	2020/5/21
39	2020/5/23	2020/5/23
40	2020/4/25	2020/5/7
41	2020/5/8	2020/5/10
42	2020/5/11	2020/5/12
43	2020/5/10	2020/5/14
44	2020/5/10	2020/5/15
45	2020/5/9	2020/5/15
46	2020/5/18	2020/5/19

The third epidemic phase

Case Number	the onset date	Date of diagnosis
1	2021/1/17	2021/1/17
2	2021/1/17	2021/1/17
3	2021/1/17	2021/1/17
4	2021/1/17	2021/1/17
5	2021/1/10	2021/1/11
6	2021/1/10	2021/1/11
7	2021/1/17	2021/1/17
8	2021/1/16	2021/1/16
9	2021/1/16	2021/1/16
10	2021/1/18	2021/1/18
11	2021/1/17	2021/1/17
12	2021/1/24	2021/1/24
13	2021/1/17	2021/1/17
14	2021/1/18	2021/1/18
15	2021/1/19	2021/1/19
16	2021/1/23	2021/1/23
17	2021/1/18	2021/1/18
18	2021/1/20	2021/1/20
19	2021/1/18	2021/1/18
20	2021/1/19	2021/1/19
21	2021/1/17	2021/1/17
22	2021/1/17	2021/1/17
23	2021/1/16	2021/1/16
24	2021/1/20	2021/1/20
25	2021/1/20	2021/1/20
26	2021/1/17	2021/1/17
27	2021/1/18	2021/1/18
28	2021/1/16	2021/1/16
29	2021/1/18	2021/1/18
30	2021/1/17	2021/1/17
31	2021/2/2	2021/2/2
32	2021/1/16	2021/1/16
33	2021/1/17	2021/1/17
34	2021/1/23	2021/1/23
35	2021/1/17	2021/1/17
36	2021/1/24	2021/1/24
37	2021/1/18	2021/1/18
38	2021/1/16	2021/1/16
39	2021/1/20	2021/1/20
40	2021/1/17	2021/1/17
41	2021/1/24	2021/1/24
42	2021/1/17	2021/1/17
43	2021/1/24	2021/1/24
44	2021/1/18	2021/1/18
45	2021/1/17	2021/1/17
46	2021/1/17	2021/1/17
47	2021/1/17	2021/1/17
48	2021/1/19	2021/1/19
49	2021/1/18	2021/1/18
50	2021/1/20	2021/1/20
51	2021/1/18	2021/1/18
52	2021/1/18	2021/1/18

53	2021/1/16	2021/1/16
54	2021/1/16	2021/1/16
55	2021/1/18	2021/1/18
56	2021/2/3	2021/2/3
57	2021/1/18	2021/1/18
58	2021/1/18	2021/1/18
59	2021/1/18	2021/1/18
60	2021/1/18	2021/1/18
61	2021/1/18	2021/1/18
62	2021/1/18	2021/1/18
63	2021/1/24	2021/1/24
64	2021/1/20	2021/1/20
65	2021/1/16	2021/1/16
66	2021/1/18	2021/1/18
67	2021/1/16	2021/1/16
68	2021/1/20	2021/1/20
69	2021/1/17	2021/1/17
70	2021/1/16	2021/1/16
71	2021/1/16	2021/1/16
72	2021/1/20	2021/1/20
73	2021/1/20	2021/1/20
74	2021/1/30	2021/1/30
75	2021/1/19	2021/1/19
76	2021/1/18	2021/1/18
77	2021/1/18	2021/1/18
78	2021/1/16	2021/1/16
79	2021/1/20	2021/1/20
80	2021/1/19	2021/1/19
81	2021/1/19	2021/1/19
82	2021/1/20	2021/1/20
83	2021/1/16	2021/1/16
84	2021/1/18	2021/1/18
85	2021/1/18	2021/1/18
86	2021/1/17	2021/1/17
87	2021/1/18	2021/1/18
88	2021/1/18	2021/1/18
89	2021/1/17	2021/1/17
90	2021/1/24	2021/1/24
91	2021/1/17	2021/1/17
92	2021/1/30	2021/1/30
93	2021/1/19	2021/1/19
94	2021/1/19	2021/1/19
95	2021/1/17	2021/1/17
96	2021/1/18	2021/1/18
97	2021/1/17	2021/1/17
98	2021/1/17	2021/1/17
99	2021/1/19	2021/1/19
100	2021/1/18	2021/1/18
101	2021/1/20	2021/1/20
102	2021/1/17	2021/1/17
103	2021/1/20	2021/1/20
104	2021/1/18	2021/1/18
105	2021/1/18	2021/1/18
106	2021/1/20	2021/1/20

107	2021/1/19	2021/1/19
108	2021/1/20	2021/1/20
109	2021/1/17	2021/1/17
110	2021/1/29	2021/1/29
111	2021/1/20	2021/1/20
112	2021/1/16	2021/1/17
113	2021/1/16	2021/1/17
114	2021/1/16	2021/1/17
115	2021/1/18	2021/1/18
116	2021/1/19	2021/1/19
117	2021/1/20	2021/1/20
118	2021/1/18	2021/1/18
119	2021/1/21	2021/1/21
120	2021/1/19	2021/1/19
121	2021/1/18	2021/1/18
122	2021/1/18	2021/1/18
123	2021/1/18	2021/1/18
124	2021/1/24	2021/1/24
125	2021/1/19	2021/1/19
126	2021/1/24	2021/1/24
127	2021/1/18	2021/1/18
128	2021/1/19	2021/1/19
129	2021/1/20	2021/1/20
130	2021/1/18	2021/1/18
131	2021/1/23	2021/1/23
132	2021/1/17	2021/1/18
133	2021/1/19	2021/1/19
134	2021/1/24	2021/1/24
135	2021/1/18	2021/1/18
136	2021/1/24	2021/1/24
137	2021/1/20	2021/1/20
138	2021/1/19	2021/1/19
139	2021/1/18	2021/1/18
140	2021/1/20	2021/1/20
141	2021/1/18	2021/1/18
142	2021/1/19	2021/1/19
143	2021/1/28	2021/1/28
144	2021/1/18	2021/1/18
145	2021/1/19	2021/1/19
146	2021/1/18	2021/1/18
147	2021/1/19	2021/1/19
148	2021/1/18	2021/1/18
149	2021/1/19	2021/1/19
150	2021/1/23	2021/1/23
151	2021/1/20	2021/1/20
152	2021/1/19	2021/1/19
153	2021/1/19	2021/1/19
154	2021/1/30	2021/1/30
155	2021/1/19	2021/1/19
156	2021/1/19	2021/1/19
157	2021/1/24	2021/1/24
158	2021/1/19	2021/1/19
159	2021/1/19	2021/1/19
160	2021/1/19	2021/1/19

161	2021/1/19	2021/1/19
162	2021/1/19	2021/1/19
163	2021/1/19	2021/1/19
164	2021/1/24	2021/1/24
165	2021/1/19	2021/1/19
166	2021/1/19	2021/1/19
167	2021/1/19	2021/1/19
168	2021/1/19	2021/1/19
169	2021/1/19	2021/1/19
170	2021/1/19	2021/1/19
171	2021/1/20	2021/1/20
172	2021/1/20	2021/1/20
173	2021/1/15	2021/1/19
174	2021/1/20	2021/1/20
175	2021/1/20	2021/1/20
176	2021/1/30	2021/1/30
177	2021/1/24	2021/1/24
178	2021/1/20	2021/1/20
179	2021/1/24	2021/1/24
180	2021/1/19	2021/1/19
181	2021/1/19	2021/1/19
182	2021/1/19	2021/1/19
183	2021/1/19	2021/1/19
184	2021/1/19	2021/1/19
185	2021/1/19	2021/1/19
186	2021/1/18	2021/1/19
187	2021/1/19	2021/1/19
188	2021/1/19	2021/1/19
189	2021/1/18	2021/1/19
190	2021/1/18	2021/1/19
191	2021/1/16	2021/1/20
192	2021/1/19	2021/1/20
193	2021/1/20	2021/1/20
194	2021/1/20	2021/1/20
195	2021/1/24	2021/1/24
196	2021/1/24	2021/1/24
197	2021/1/20	2021/1/20
198	2021/1/19	2021/1/20
199	2021/1/24	2021/1/24
200	2021/1/28	2021/1/28
201	2021/1/24	2021/1/24
202	2021/1/16	2021/1/21
203	2021/1/19	2021/1/21
204	2021/1/17	2021/1/21
205	2021/1/18	2021/1/21
206	2021/1/19	2021/1/21
207	2021/1/28	2021/1/28
208	2021/1/20	2021/1/21
209	2021/1/20	2021/1/21
210	2021/1/20	2021/1/21
211	2021/1/20	2021/1/21
212	2021/1/23	2021/1/23
213	2021/1/20	2021/1/21
214	2021/1/20	2021/1/21

215	2021/1/21	2021/1/21
216	2021/1/20	2021/1/21
217	2021/1/30	2021/1/30
218	2021/1/24	2021/1/24
219	2021/1/24	2021/1/24
220	2021/1/20	2021/1/21
221	2021/1/20	2021/1/21
222	2021/1/20	2021/1/21
223	2021/1/20	2021/1/21
224	2021/1/20	2021/1/21
225	2021/1/20	2021/1/21
226	2021/1/30	2021/1/30
227	2021/1/24	2021/1/24
228	2021/1/24	2021/1/24
229	2021/1/20	2021/1/22
230	2021/1/21	2021/1/22
231	2021/1/21	2021/1/22
232	2021/1/21	2021/1/22
233	2021/1/24	2021/1/24
234	2021/1/24	2021/1/24
235	2021/1/21	2021/1/22
236	2021/1/22	2021/1/22
237	2021/1/23	2021/1/23
238	2021/1/22	2021/1/22
239	2021/1/22	2021/1/22
240	2021/1/23	2021/1/23
241	2021/2/2	2021/2/2
242	2021/1/24	2021/1/24
243	2021/1/21	2021/1/22
244	2021/1/29	2021/1/29
245	2021/1/24	2021/1/24
246	2021/1/24	2021/1/24
247	2021/1/28	2021/1/28
248	2021/1/30	2021/1/30
249	2021/1/21	2021/1/22
250	2021/1/24	2021/1/24
251	2021/1/25	2021/1/25
252	2021/1/30	2021/1/30
253	2021/1/24	2021/1/24
254	2021/1/31	2021/1/31
255	2021/1/24	2021/1/24
256	2021/1/24	2021/1/24
257	2021/1/28	2021/1/28
258	2021/1/25	2021/1/25
259	2021/1/25	2021/1/25
260	2021/1/24	2021/1/24
261	2021/1/24	2021/1/24
262	2021/1/30	2021/1/30
263	2021/1/24	2021/1/24
264	2021/1/24	2021/1/24
265	2021/2/3	2021/2/3
266	2021/1/30	2021/1/30
267	2021/1/31	2021/1/31
268	2021/1/25	2021/1/25

269	2021/1/24	2021/1/24
270	2021/1/24	2021/1/24
271	2021/1/25	2021/1/25
272	2021/1/24	2021/1/24
273	2021/1/25	2021/1/25
274	2021/1/30	2021/1/30
275	2021/1/30	2021/1/30
276	2021/1/22	2021/1/22
277	2021/1/24	2021/1/24
278	2021/1/24	2021/1/24
279	2021/1/25	2021/1/25
280	2021/1/30	2021/1/30
281	2021/1/19	2021/1/22
282	2021/1/22	2021/1/22
283	2021/1/20	2021/1/22
284	2021/1/30	2021/1/30
285	2021/1/22	2021/1/22
286	2021/1/30	2021/1/30
287	2021/1/24	2021/1/24
288	2021/1/24	2021/1/24
289	2021/1/24	2021/1/24
290	2021/1/24	2021/1/24
291	2021/1/22	2021/1/22
292	2021/1/22	2021/2/2
293	2021/1/24	2021/1/24
294	2021/1/22	2021/1/23
295	2021/1/30	2021/1/30
296	2021/1/24	2021/1/24
297	2021/1/24	2021/1/24
298	2021/1/24	2021/1/24
299	2021/1/24	2021/1/24
300	2021/1/23	2021/1/23
301	2021/1/23	2021/1/23
302	2021/1/24	2021/1/24
303	2021/1/24	2021/1/24
304	2021/1/24	2021/1/24
305	2021/1/24	2021/1/24
306	2021/1/24	2021/1/24
307	2021/1/24	2021/1/24
308	2021/1/24	2021/1/24
309	2021/1/30	2021/1/30
310	2021/1/31	2021/1/31
311	2021/1/24	2021/1/24
312	2021/1/24	2021/1/24
313	2021/1/30	2021/1/30
314	2021/1/24	2021/1/24
315	2021/1/24	2021/1/24
316	2021/1/30	2021/1/30
317	2021/1/24	2021/1/24
318	2021/1/23	2021/1/23
319	2021/1/24	2021/1/24
320	2021/2/3	2021/2/3
321	2021/1/23	2021/1/23
322	2021/1/30	2021/1/30

323	2021/1/23	2021/1/23
324	2021/1/23	2021/1/23
325	2021/1/23	2021/1/23
326	2021/1/24	2021/1/24
327	2021/1/23	2021/1/24
328	2021/1/30	2021/1/30
329	2021/1/30	2021/1/30
330	2021/1/26	2021/1/26
331	2021/1/24	2021/1/24
332	2021/1/24	2021/1/24
333	2021/1/26	2021/1/26
334	2021/1/30	2021/1/30
335	2021/1/30	2021/1/30
336	2021/1/30	2021/1/30
337	2021/1/30	2021/1/30
338	2021/1/30	2021/1/30
339	2021/1/30	2021/1/30
340	2021/2/1	2021/2/1
341	2021/1/30	2021/1/30
342	2021/1/30	2021/1/30
343	2021/1/30	2021/1/30
344	2021/1/28	2021/1/28
345	2021/1/30	2021/1/30
346	2021/1/30	2021/1/30
347	2021/1/30	2021/1/30
348	2021/1/30	2021/1/30
349	2021/1/25	2021/1/26
350	2021/1/25	2021/1/26
351	2021/1/24	2021/1/26
352	2021/1/25	2021/1/26
353	2021/1/26	2021/1/26
354	2021/1/26	2021/1/26
355	2021/1/26	2021/1/26
356	2021/1/26	2021/1/26
357	2021/1/30	2021/1/30
358	2021/1/28	2021/1/28
359	2021/1/30	2021/1/30
360	2021/1/30	2021/1/30
361	2021/2/3	2021/2/3
362	2021/1/30	2021/1/30
363	2021/1/30	2021/1/30
364	2021/2/2	2021/2/2
365	2021/1/30	2021/1/30
366	2021/1/30	2021/1/30
367	2021/1/30	2021/1/30
368	2021/1/30	2021/1/30
369	2021/1/27	2021/1/27
370	2021/1/27	2021/1/27
371	2021/2/2	2021/2/2
372	2021/1/31	2021/1/31
373	2021/1/23	2021/1/26
374	2021/1/24	2021/1/26
375	2021/1/24	2021/1/26
376	2021/1/30	2021/1/30

377	2021/1/23	2021/1/26
378	2021/1/30	2021/1/30
379	2021/1/26	2021/1/27
380	2021/1/30	2021/1/30
381	2021/1/29	2021/1/29
382	2021/1/29	2021/1/29
383	2021/1/30	2021/1/30
384	2021/1/27	2021/1/27
385	2021/1/27	2021/1/27
386	2021/1/27	2021/1/27
387	2021/1/27	2021/1/27
388	2021/1/27	2021/1/27
389	2021/2/1	2021/2/1
390	2021/2/1	2021/2/1
391	2021/1/28	2021/1/28
392	2021/1/27	2021/1/27
393	2021/1/27	2021/1/27
394	2021/1/30	2021/1/30
395	2021/1/28	2021/1/28
396	2021/1/21	2021/1/27
397	2021/1/22	2021/1/28
398	2021/1/30	2021/1/30
399	2021/1/30	2021/1/30
400	2021/2/2	2021/2/2
401	2021/1/30	2021/1/30
402	2021/1/28	2021/1/28
403	2021/1/28	2021/1/28
404	2021/1/30	2021/1/30
405	2021/1/30	2021/1/30
406	2021/1/30	2021/1/30
407	2021/1/28	2021/1/29
408	2021/1/30	2021/1/30
409	2021/1/30	2021/1/30
410	2021/1/17	2021/1/31
411	2021/1/29	2021/1/30
412	2021/1/29	2021/1/30
413	2021/1/30	2021/1/30
414	2021/1/30	2021/1/30
415	2021/1/31	2021/1/31
416	2021/1/29	2021/1/30
417	2021/1/25	2021/1/30
418	2021/1/31	2021/1/31
419	2021/1/31	2021/1/31
420	2021/1/30	2021/1/31
421	2021/1/30	2021/1/31
422	2021/2/1	2021/2/1
423	2021/2/3	2021/2/3
424	2021/2/2	2021/2/2
425	2021/2/2	2021/2/2
426	2021/2/2	2021/2/2
427	2021/2/3	2021/2/3
428	2021/2/2	2021/2/3
429	2021/2/3	2021/2/3
430	2021/2/3	2021/2/4

431	2021/2/4	2021/2/4
432	2021/2/6	2021/2/6
433	2021/2/5	2021/2/5
434	2021/2/5	2021/2/6
435	2021/2/9	2021/2/10
